# Paxillin is a potential prognostic biomarker associated with immune cell infiltration in ovarian cancer

**DOI:** 10.1016/j.heliyon.2023.e14095

**Published:** 2023-02-27

**Authors:** Li-qun Meng, Ling-yan Zhang, Wen-zhi Xu

**Affiliations:** a Operating Room, Sir Run Run Shaw Hospital, Zhejiang University School of Medicine, Hangzhou 310016, Zhejiang, China; b Department of Obstetrics and Gynecology, Sir Run Run Shaw Hospital, Zhejiang University School of Medicine, Hangzhou 310016, Zhejiang, China

**Keywords:** Paxillin, Ovarian cancer, Prognostic biomarker, Immune cell infiltration

## Abstract

**Objective:**

To investigate the expression, prognosis, and underlying mechanism of Paxillin (PXN) in ovarian cancer.

**Materials and methods:**

By comprehensive use of various bioinformatics tools, we analyzed the expression of PXN and its prognostic value in ovarian cancer. Then, the enrichment analyses were conducted to determine the possible regulatory pathways PXN involved in ovarian cancer. Finally, the associations of PXN expression with immune cell infiltration and immune checkpoints were analyzed.

**Results:**

PXN was highly expressed in ovarian cancer and its expression could independently predict the overall survival of ovarian cancer patients. More importantly, PXN had a superior ability in predicting long-term survival than age and tumor residual disease in ovarian cancer patients. In addition, PXN was positively related to adherens junction and tight junction pathways. Significant negative relationships between PXN expression and immune infiltrates were observed, however, PXN was positively connected with immune checkpoint (VSIR) in ovarian cancer.

**Conclusions:**

PXN serves as a reliable prognostic biomarker and may be a potent therapeutic target for ovarian cancer. Moreover, high PXN expression may affect ovarian cancer progression via positive regulation of metastasis-related pathways.

## 1 Introduction

Ovarian cancer is the most lethal gynecological malignancy seriously affecting women's health and life worldwide [1]. It was estimated that 308,069 cases of ovarian cancer would be diagnosed and 193,811 patients would succumb to this disease in 2020 globally [2]. In the United States, approximately 22,000 incident cases and 14,000 deaths due to ovarian cancer each year, which represents to be the fifth most predominant cause of cancer mortality in women [3]. Most patients (70%) suffering from ovarian cancer are diagnosed at an advanced stage due to occult onset, rapid progression, and the absence of typical early symptoms [4]. Debulking surgery and platinum-taxane maintenance chemotherapy are the front-line standard treatment for ovarian cancer [5]. The response rate to first-line therapy is around 80–90%, but the five-year survival rate of ovarian cancer is still unsatisfactory owing to relapse and chemoresistance [6]. Besides, long-term survival which is defined as living 10 years or longer after diagnosis remains relatively low [7]. Currently, several serum biomarkers such as BRAC1, HE4, and CA125 were used as diagnostic tools for ovarian cancer [[8], [9], [10]]. However, these indicators are not ideal for evaluating the prognosis of patients [11]. There is an obvious need to develop novel biomarkers for improving the prognosis of ovarian cancer patients.

Paxillin (PXN), a structural protein of 68 kDa, is localized to human chromosome 12q24.31 and is originally identified as a tyrosine-phosphorylated protein in chick embryo fibroblasts [12]. It represents a prominent role in the transduction of extracellular signals to intracellular responses, triggered by the binding of integrins to the extracellular matrix [13]. PXN is actively involved in the reorganization of the actin cytoskeleton, signals integration, and the assembly/disassembly of focal adhesions, which are required for maintaining cell morphology, cell motility, proliferation and survival, cell apoptosis, metastasis, and angiogenesis [14]. Previous studies have demonstrated the involvement of PXN in multiple cancers. PXN promoted tumor progression and predicted survival and relapse in oral cavity squamous cell carcinoma [15]. Besides, upregulated PXN expression was observed in gastric cancer, and its abnormal expression correlated with tumor progression and poor prognosis [16]. Kawada et al. reported that PXN was overexpressed in lung cancer patient samples and the mutant PXN variants might be implicated in lung cancer progression, which might be a therapeutic target [17]. However, there is a lack of systemic research on the expression and the prognostic role of PXN in ovarian cancer.

In this study, we evaluated the PXN expression in ovarian cancer and its association with patient prognosis. We also analyzed the role of PXN expression in predicting long-term survival. Then, enrichment analyses were conducted to explore the underlying mechanism of PXN in ovarian cancer. The association of PXN expression with immune cell infiltrates and immune checkpoints was assessed to exhibit the possible value of PXN in immunotherapy. Finally, we explored the chemicals interacting with the PXN gene.

## 2 Materials and methods

### 2.1 Expression analysis of PXN

Due to the absence of matched normal samples in the TCGA database, the differential PXN expressions in pan-cancer and normal tissues were analyzed based on data from TCGA and GTEx. The data were used to extract the mRNA expression values of PXN and the tumors with less than three samples were deleted. 10.13039/100000085Gene Expression Omnibus (GEO) (https://www.ncbi.nlm.nih.gov/geo/) is a public functional genomics data repository supporting MIAME-compliant data submissions. According to the following inclusion criteria: with more than 45 samples; containing ovarian cancer and normal ovary samples; including expression data, the GSE18520 (including 53 tumor and 10 normal samples) and GSE27651 datasets (containing 43 tumor and 6 normal samples) were obtained to evaluate the PXN expression in ovarian cancer and normal ovary tissue. The HPA database (https://www.proteinatlas.org/) aims to map all the human proteins in cells, tissues, and organs using an integration of various omics technologies. We used this database to examine the protein level of PXN in ovarian cancer and normal ovary tissues by immunohistochemical (IHC) analysis. For protein expression analysis, in HPA, sections from cancer tissue microarrays were immunohistochemically stained and corresponding slides were scanned to generate digital images. All images were then analyzed by pathologists and annotated with respect to staining intensity and fraction of positive cancer cells for all approved antibodies. The result of immunohistochemistry-based protein expression was then summarized as high, medium, low or not detected. Moreover, we adopted Image J software to quantify the PXN protein in ovarian cancer and normal ovary tissues. In addition, we conducted IHC analysis to verify the PXN protein level in ovarian cancer tissue and normal ovary tissue following the manufacturer's protocols.

Then, the expression data and corresponding clinical information in GDC TCGA-ovarian cancer were downloaded from the UCSC Xena database (https://xenabrowser.net/datapages/). Only 378 patients with matched expression and survival information were enrolled. Patients were divided into high and low PXN expression groups based on the median level of PXN. The association of PXN expression with clinicopathological characteristics was assessed using the TCGA-ovarian cancer data.

### 2.2 Prognostic analysis of PXN

The relationship between PXN expression and overall survival (OS) was analyzed using the GDC TCGA-ovarian cancer data using Kaplan-Meier method. Patients were separated into two groups with the best cutoff [18]. Next, the GSE63885 dataset (n = 70) was acquired to validate the effect of PXN expression on the OS of ovarian cancer patients. The inclusion criteria were as follows: containing over 45 ovarian cancer samples; including expression data; with survival data.

Following this, both univariate and multivariate Cox regression analyses were performed to investigate the independent factors for predicting the OS of ovarian cancer patients based on the GDC TCGA-ovarian cancer data and GSE63885 data.

Subsequently, the value of PXN and other clinical variables in distinguishing long-term survival (≥10 years) from short-term survival (<3 years) was assessed by calculating the area under the curve (AUC) of the receiver operating characteristic (ROC) curve. Then, the “rms” in the R package was adopted to construct a nomogram to predict long-term survival for ovarian cancer patients.

### 2.3 Enrichment analyses

The genes co-expressed with PXN were identified using the cBioportal database (https://www.cbioportal.org/). We selected ovarian serous cystadenocarcinoma (TCGA, Firehose Legacy) and clicked “Query by Gene”. Then, we entered the “PXN” gene and chose the “Co-expression” column. Totally 19,923 co-expressed genes were shown and finally, 631 genes with high correlation were screened out for functional enrichment analysis with a q-value <0.01 and an absolute value of Spearman's R > 0.03 as a threshold. Gene ontology annotations, and Kyoto Encyclopedia of Genes and Genomes (KEGG) pathway analysis of PXN and its co-expressed genes were performed using the “clusterProfiler” in R.

Further, the gene set enrichment analysis (GSEA) was performed to illustrate the significant survival difference between the high- and low- PXN expression groups. The gene set was permutated 1000 times and the expression level of PXN was used as a phenotypic label. A nominal p-value <0.05 and a false discovery rate q-value were considered to be statistically significant.

Following this, we defined the enrichment level of a pathway in ovarian cancer samples as the single-sample gene-set enrichment analysis (ssGSEA) score [19]. The gene set represents the collection of all marker genes of a pathway. The association of the PXN mRNA expression with the enrichment levels (ssGSEA scores) of the pathway was evaluated using Pearson's correlation test.

### 2.4 Immune infiltration analysis

To evaluate the correlation between PXN expression and tumor immune microenvironment in ovarian cancer, the TIMER database (http://timer.comp-genomics.org/) was adopted to analyze the association of PXN expression with infiltration levels of B cell, CD8 + T cell, CD4 + T cell, macrophage, neutrophil, and dendritic cell in ovarian cancer. TIMER database includes 10,897 samples from 32 cancer types in TCGA and pre-calculates the infiltration levels of six types of immune cells. Moreover, the relationship between PXN expression and immune checkpoint levels was evaluated using the Pearson correlation test in the TCGA-ovarian cancer cohort. Additionally, the RNA-seq expression profile data and clinical information of ovarian cancer patients were downloaded from the ICGC database and OV-AU (Ovarian cancer-Australia) dataset was obtained for subsequent validation.

### 2.5 Statistical analysis

All statistical analyses were performed using SPSS software (SPSS, Inc., Chicago, IL, USA), and R software. The *t*-test was used to analyze differences in each two-group comparison, and one-way ANOVA was employed to assess differences among at least three groups. The association of PXN expression as a categorical variable with clinicopathological characteristics was analyzed by the chi-square test. Survival curves were drawn by the Kaplan-Meier method and differences in survival were compared by log-rank tests. P < 0.05 was considered statistically significant.

## 3 Results

### 3.1 High PXN expression in ovarian cancer

**Fig. 1:**
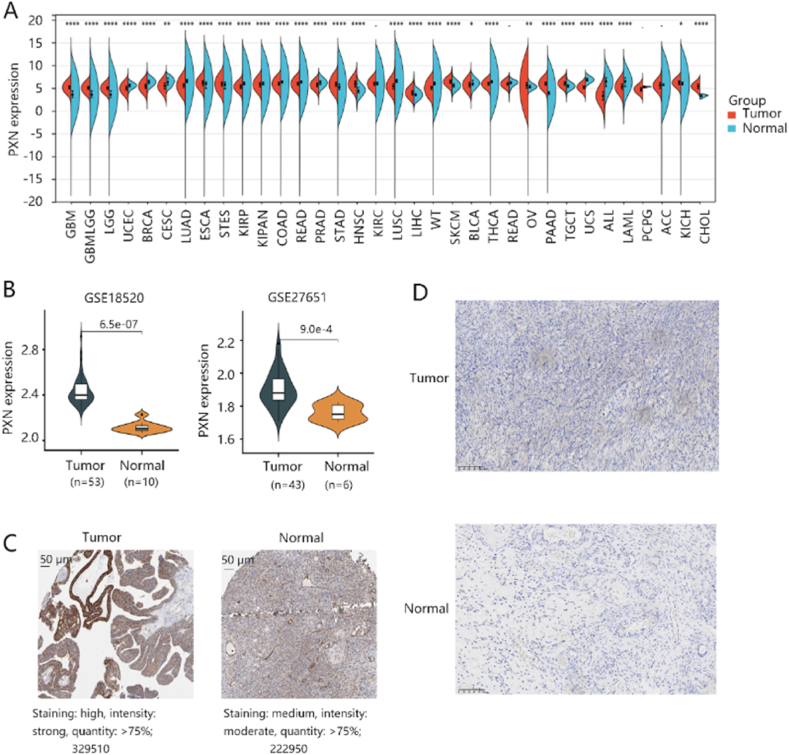
The expression profile of PXN expression in tumors. **(A)** The differential PXN expression in various cancers and normal tissues based on TCGA and GTEx data. **(B)** The elevated mRNA level of PXN in the ovarian cancer group compared with the normal ovary group using the GSE18520 and GSE27651 datasets. **(C)** The PXN protein level in ovarian cancer and normal ovary tissues from the HPA database. Image J software was used to quantify the PXN protein in ovarian cancer and normal tissues. (D) The protein expression of PXN in the ovarian cancer and normal ovary tissues by the IHC analysis.

To have an overview of PXN expression, we first analyzed the expression of PXN in various cancers as shown in Fig. 1A. Of note, PXN mRNA expression was significantly higher in ovarian cancer tissue compared with that in normal ovary tissue in both GSE18520 and GSE27651 datasets (all P < 0.05) (Fig. 1B). IHC analysis showed that higher protein level of PXN in ovarian cancer tissue than normal ovary tissue (Fig. 1C–D). These results indicated PXN upregulation in ovarian cancer tissue.

**Fig. 2:**
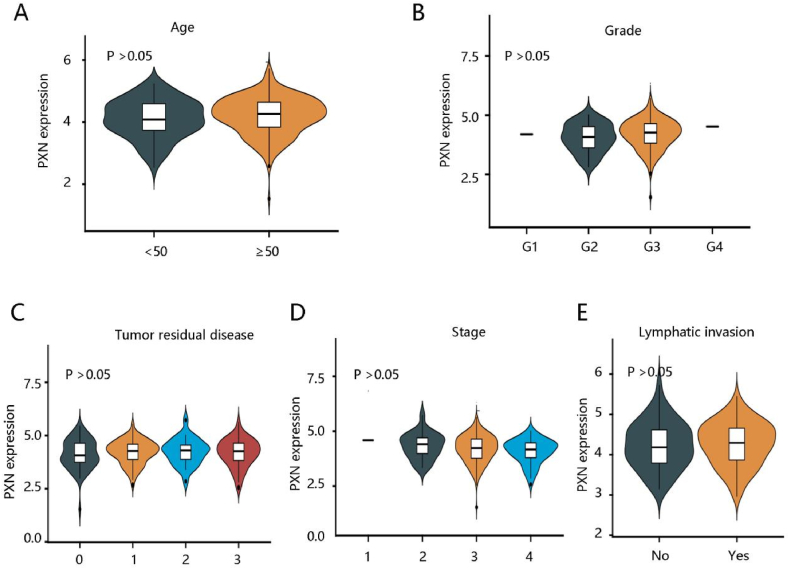
The relationship between PXN expression and clinicopathological characteristics in ovarian cancer. **(A)** Age. **(B)** Grade. **(C)** Tumor residual disease. **(D)** Stage. **(E)** Lymphatic invasion.

Then, the distribution of age, grade, tumor residual disease, stage, and lymphatic invasion in low and high PXN expression groups was evaluated using the chi-square test. Interestingly, there was no significant distribution of age, grade, tumor residual disease, stage, and lymphatic invasion in the two expression groups (all P > 0.05) (Table 1). Similarly, PXN expression as a continuous variable was also not significantly related to age, grade, tumor residual disease, stage and lymphatic invasion (all P > 0.05) (Fig. 2A–E).

**Table 1:** The relationship between PXN expression and clinicopathological characteristics in ovarian cancer.

Characteristics	Low PXN	High PXN	P value
Age			0.380
<50	44	37	
≥50	145	152	
Grade			0.069
1	1	0	
2	29	16	
3	154	167	
4	0	1	
Tumor residual disease			0.386
No macroscopic disease	39	28	
1–10 mm	79	92	
11–20 mm	12	15	
>20 mm	34	36	
Stage			0.663
1	0	1	
2	11	12	
3	147	147	
4	30	27	
Lymphatic invasion			0.296
No	26	22	
Yes	45	56	
Unknown	118	111

### 3.2 High PXN expression independently predicted poor OS of ovarian cancer patients

**Fig. 3:**
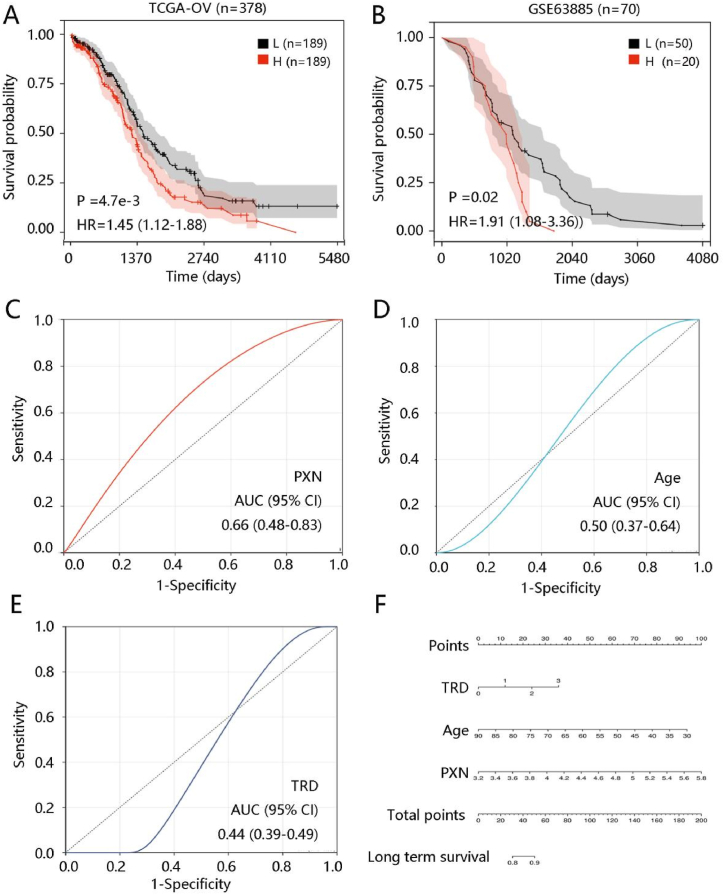
The clinical value of PXN in ovarian cancer. High PXN expression predicted unfavorable overall survival in ovarian cancer patients. **(A)** TCGA dataset. **(B)** GSE63885 dataset. The value of **(C)** PXN, **(D)** age, and **(E)** tumor residual disease in predicting long-term survival. **(F)** Nomogram generation including tumor residual disease, age, and PXN. **Abbreviations:** OV, ovarian cancer; HR, hazard ratio; AUC, area under the curve; 95% CI, 95% confidence interval; TRD, tumor residual disease.

To determine the prognostic value of PXN in ovarian cancer, Kaplan-Meier curves were drawn to analyze the correlation of PXN expression with OS using the TCGA data and GSE63885 dataset. As shown in Fig. 3A–B, ovarian patients with high PXN expression had significantly shorter OS (all P < 0.05). Further stratified analysis showed that high PXN expression led to the unfavorable OS in patients aged ≥50 years, grades 3 + 4, tumor residual disease >20 mm, and stage 3 + 4 (P < 0.05) (Table 2).

**Table 2:** Stratified analysis of PXN on clinicopathological parameters in ovarian cancer.

Parameters	Hazard ratio	95% confidence interval	P value
Age			
<50	2.42	1.18–4.96	0.01
≥50	1.49	1.12–1.97	6.0e-3
Grade			
1 + 2	1.89	0.79–4.54	0.15
3 + 4	1.51	1.13–2.00	4.3e-3
Tumor residual disease			
≤20 mm	1.35	0.99–1.85	0.06
>20 mm	2.32	1.25–4.29	5.9e-3
Stage			
1 + 2	3.28	0.60–17.95	0.15
3 + 4	1.46	1.12–1.90	4.4e-3

To investigate the independent prognostic factors for predicting OS of ovarian cancer patients, age, grade, tumor residual disease, stage, and PXN were included in Cox regression analysis in the TCGA-ovarian cancer cohort. In the univariate analysis, age, tumor residual disease, and PXN were significantly related to OS (P < 0.05). The multivariate analysis showed that age, tumor residual disease, and PXN independently predicted the OS of ovarian cancer patients (P < 0.05) (Table 3). For validation, we performed univariate and multivariate Cox regression analysis using the GSE63885 dataset. Consistently, PXN remained an independent prognostic factor for patients with ovarian cancer (Table 4). These results indicated that PXN expression could be an independent prognostic factor for OS among ovarian cancer patients.

**Table 3:** Cox regression analysis of PXN and clinical variables for overall survival in ovarian cancer using the TCGA cohort.

Characteristics	Univariate analysis		Multivariate analysis	
	HR (95% CI)	P value	HR (95% CI)	P value
Age	1.021 (1.009–1.034)	0.001	1.019 (1.006–1.033)	0.004
Grade	1.252 (0.853–1.838)	0.251	1.267 (0.834–1.926)	0.267
Tumor residual disease	1.312 (1.150–1.496)	<0.001	1.259 (1.098–1.444)	0.001
Stage	1.312 (0.987–1.744)	0.062	1.325 (0.963–1.823)	0.084
PXN	1.279 (1.033–1.583)	0.024	1.276 (1.010–1.611)	0.041

**Abbreviations:** HR, hazard ratio; 95% CI, 95% confidence interval.

**Table 4:** Cox regression analysis of PXN and clinical variables for overall survival in ovarian cancer using the GSE63885 data.

Characteristics	Univariate analysis		Multivariate analysis	
	HR (95% CI)	P value	HR (95% CI)	P value
Stage	1.918 (0.982–3.744)	0.056	1.256 (0.609–2.592)	0.537
Tumor residual disease	1.723 (1.230–2.413)	0.002	1.503 (1.050–2.151)	0.026
Grade	1.374 (0.910–2.075)	0.130	1.561 (1.031–2.364)	0.035
Post front-line chemotherapy	1.983 (1.507–2.611)	<0.001	1.965 (1.448–2.666)	<0.001
PXN	2.156 (1.093–4.253)	0.027	2.060 (1.062–3.998)	0.033

**Abbreviations:** HR, hazard ratio; 95% CI, 95% confidence interval.

Further, we explored the value of the three independent variables in distinguishing long-term survival and short-term survival among ovarian cancer patients in the TCGA dataset. PXN expression presented a superior ability in predicting long-term survival compared with age and tumor residual disease. The AUCs were 0.66, 0.50, and 0.44 for PXN, age, and tumor residual disease, respectively (Fig. 3C–E). The three factors were included in the nomogram construction and PXN had the highest contribution (Fig. 3F).

### 3.3 PXN is mainly involved in metastasis-related pathways

**Fig. 4:**
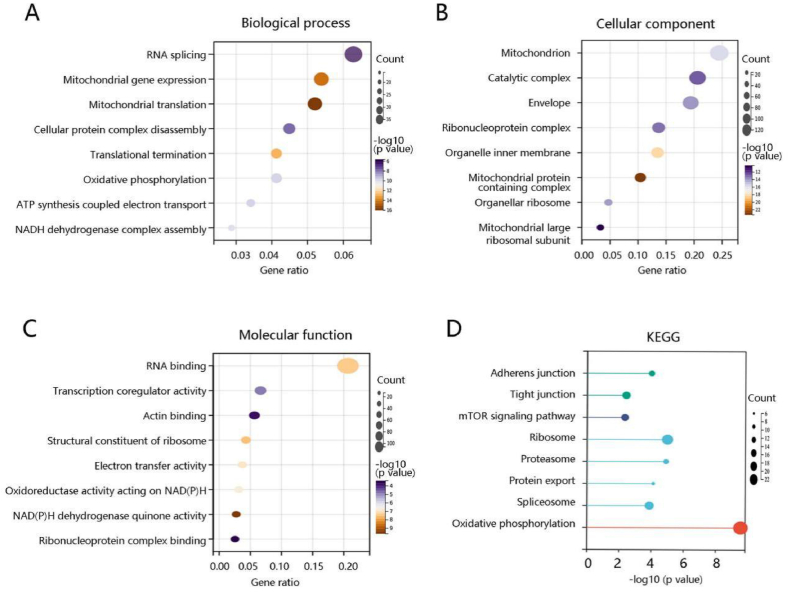
Functional enrichment analysis of the PXN gene and its co-expressed genes. The **(A)** biological process, **(B)** cellular component, **(C)** molecular function, and **(D)** KEGG pathways of these genes.

To elucidate the pathological role of PXN in ovarian cancer, we performed a functional enrichment analysis of PXN and its co-expressed genes. As for biological processes, these genes were mainly involved in RNA splicing, mitochondrial gene expression, and mitochondrial translation (Fig. 4A). The major cellular components were mitochondrion, catalytic complex, and envelope (Fig. 4B). In terms of molecular functions, they mainly participated in RNA binding, transcription coregulatory activity, and actin binding (Fig. 4C). The potential KEGG pathways were adherens junction, tight junction, mTOR signaling pathway, ribosome, and proteasome (Fig. 4D).

**Fig. 5:**
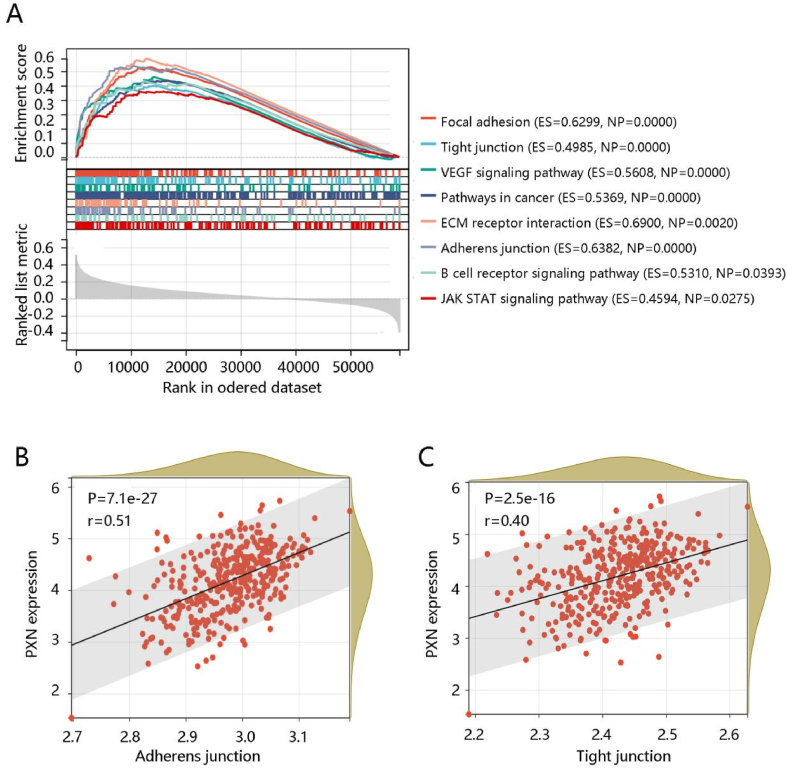
The major pathways enriched in high PXN expression group. **(A)** The pathways enriched in the high PXN expression group. PXN expression was positively related to the enrichment levels of **(B)** adherens junction and **(C)** tight junction. Abbreviations: ES, enrichment score; NP, nominal P.

To further reveal the underlying mechanism of PXN in ovarian cancer, GSEA was performed using the TCGA data. Adherens junction, focal adhesion, pathways in cancer, VEGF signaling pathway, tight junction, ECM receptor interaction, B cell receptor signaling pathway, and JAK STAT signaling pathway were mainly enriched in the high PXN expression group (Fig. 5A). Combined with the previous enrichment analysis results, PXN expression mainly regulated adherens junction and tight junction pathways.

Moreover, we analyzed the enrichment levels of the adherens junction and tight junction pathways using ssGSEA. The PXN gene expression level had a significant positive correlation with the enrichment levels (ssGSEA scores) of the adherens junction and tight junction pathways (all P < 0.001) (Fig. 5B–C). The above findings suggested that PXN might affect ovarian cancer progression through positive regulation of adherens junction and tight junction pathways.

### 3.4 PXN was negatively related to immune cell infiltrates

**Fig. 6:**
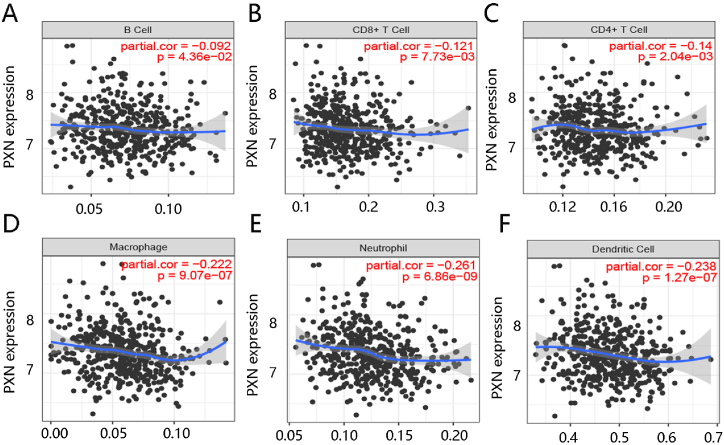
The possible immune function of PXN in ovarian cancer. The negative association of PXN expression with six immune cells including **(A)** B cell, **(B)** CD8^+^ T cell, **(C)** CD4^+^ T cell, **(D)** Macrophage, **(E)** Neutrophil and **(F)** Dendritic cell.

**Fig. 7:**
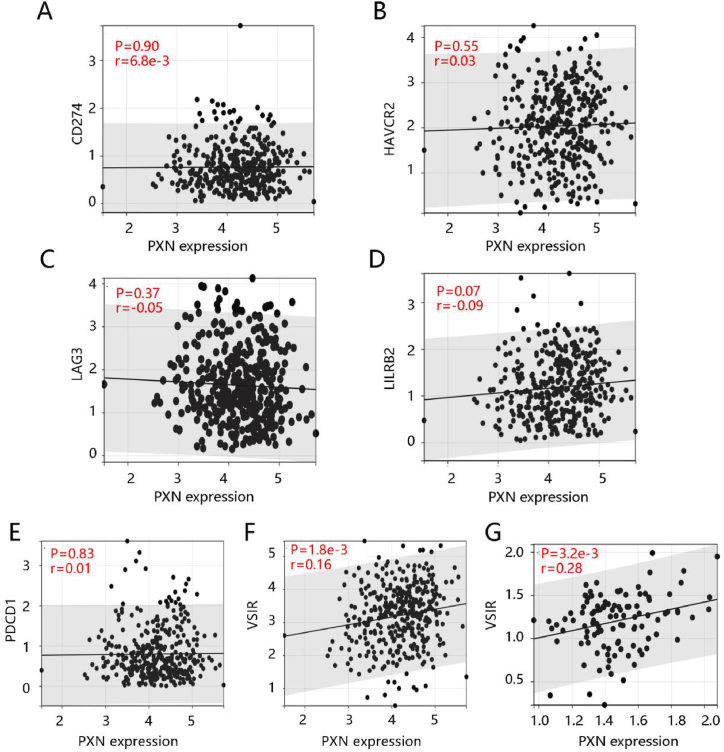
The relationship between PXN expression with the expression levels of six immune checkpoints including **(A)** CD274, **(B)** HAVCR2, **(C)** LAG3, **(D)** LILRB2, **(E)** PDCD1, and **(F)** VSIR using the TCGA data. **(G)** ICGC data confirmed the significant relationship between PXN and VSIR.

Due to the essential role of the tumor immune microenvironment in ovarian cancer, we assessed the correlation of PXN expression with infiltration levels of six tumor immune cells using the TCGA data. Fig. 6A–F presents that PXN expression had a close negative relation with B cell, CD8^+^ T cell, CD4^+^ T cell, macrophage, neutrophil, and dendritic cell infiltration levels (all P < 0.05). Besides, we explored the association of PXN expression levels with several immune checkpoints’ expression. Although PXN expression was not significantly linked to the expression levels of CD274, HAVCR2, LAG3, LILRB2, and PDCD1, it was positively connected with VSIR expression (Fig. 7A–F). The ICGA data further confirmed the significant association of PXN with VSIR (P < 0.05) (Fig. 7G). These results indicated that PXN might play a certain role in immunotherapy in ovarian cancer.

## 4 Discussion

Ovarian cancer is an aggressive gynecological malignancy with a 5-year survival rate below 45% [20]. Although the advancement in diagnosis and treatment has been achieved, ovarian cancer remains a serious threat to women's health and a salient public concern. With the development of high-throughput sequencing technology and transcriptomic research, an increasing number of key driver genes such as CDC20 and FCGBP have been identified [21,22]. Nevertheless, there remains an urgent need to explore additional potent biomarkers, especially those related to the immune microenvironment to improve the prognosis of ovarian cancer patients. PXN encodes a cytoskeletal protein that involves actin-membrane attachment to the extracellular matrix [23]. It has been reported that PXN overexpression promoted colorectal cancer progression and metastasis [24]. Functional links between PXN and tumorigenesis such as renal cell carcinoma, breast cancer, and glioblastoma have been revealed [[25], [26], [27]]. Our study demonstrated the potential function of PXN in ovarian cancer.

Through expression analysis, we found that PXN was highly expressed in ovarian cancer, but its expression was not significantly related to age, grade, tumor residual disease, and stage. Using the TCGA-ovarian cancer and GSE63885 datasets, we drew Kaplan-Meier plotter curves and observed that patients with high PXN expression had worse clinical outcomes. Cox regression analysis revealed PXN, age, and tumor residual disease as independent prognostic biomarkers for ovarian cancer. Among them, PXN exhibited satisfactory performance compared with age, and tumor residual disease in predicting long-term survival of ovarian cancer patients.

Further, we explored the possible underlying mechanism of PXN in ovarian cancer. Various studies have demonstrated that PXN enhances cancer proliferation through the activation of multiple signaling pathways. In non-small cell lung cancer cells, PXN activated ERK and increased the binding of CREB to the Bcl-2 promoter, elevating the Bcl-2 expression level and reducing cellular apoptosis [28]. Phosphorylation of PXN activated ERK/ELK1 and increased the transcription of c-FOS and cyclin D1, which in turn facilitated the proliferation of prostate cancer cells [29]. As the main component of focal adhesions, PXN might improve the adhesive abilities of cancer cells toward the nearby stroma and matrix, hence contributing to tumor adhesion and migration [30]. Herein, the enrichment analysis result showed that PXN was mainly involved in adherens junction, focal adhesion, and tight junction pathways. Epithelial and endothelial cells provide protective barriers including tight junction and adherens junction for cells in most living systems, shielding various organs from the surrounding environment and helping to maintain homeostasis [31,32]. A tight junction primarily acts as a barrier to solutes and water and as a fence that separates the apical and basolateral plasma membrane domain [33]. Adherens junction provides mechanical adhesion and is responsible for binding with the cytoskeleton [34]. Both junctions are responsible for maintaining/imparting cell polarity [35]. Dysregulation of cell junction adhesion was implicated in the process of epithelial-mesenchymal transition, promoting cancer cell migration and invasion [36]. Therefore, the authors speculated that PXN might affect the poor prognosis of ovarian cancer patients through activation of these pathways and accelerating cancer metastasis.

Ovarian cancer is considered to be one of the most immunogenic tumors [37]. Immunotherapy has attracted extensive attention and has shown promising potential in the treatment of ovarian cancer [38]. The immune cell infiltration analysis result showed that PXN expression was related to B cells, CD8^+^ T cells, CD4^+^ T cells, macrophages, neutrophils, and dendritic cells with statistical significance. PXN expression is increased during M2 macrophage activation. In the nude mouse model, co-injection of colon cancer cells with wild-type or PXN-depleting macrophages significantly reduced the tumor size, suggesting that PXN can modulate the immune response and promote tumor growth [39]. Besides, the abnormal PXN expression would affect the ability of T cells in lysing tumor cells and in releasing the anti-tumor cytokine INFγ [40]. The immune checkpoint blockade therapy inhibiting negative regulatory immune checkpoints through various immune checkpoint inhibitors has shown great promise for ovarian cancer treatment [41,42]. This study revealed the negative relationship between PXN expression and immune checkpoint VSIR that can act as a receptor or ligand in cellular process. It has been demonstrated that upregulated VSIR on the tumor-infiltrating myeloid cells promotes tumor growth via suppressing T cell immunity [43], indicating that PXN might exhibit certain functions in immunotherapy for ovarian cancer.

For strengths, our study comprehensively investigates the PXN expression and prognosis in ovarian cancer. Importantly, this is the first research that reveals the value of PXN in distinguishing long-term survival from short-term survival and its association with immune cell infiltration. For limitations, our study only conducts IHC analysis and lacks of other experiments. Although PXN proved to be valuable in determining prognosis and guiding treatment in ovarian cancer patients, it should be prospectively confirmed by large-sample clinical studies. Besides, we lack of the experiment to validate the potential involvement of PXN in immune cell infiltration, which should be verified in cell lines or animal models in the future.

In conclusion, PXN is highly expressed in ovarian cancer compared with normal tissues. Besides, PXN can independently predict the OS of ovarian cancer patients and exhibit relatively satisfactory performance in predicting long-term survival. Moreover, our findings provide new ideas to leverage the future development of new therapeutic agents.

## Declarations

### Availability of data and materials

The dataset used and/or analyzed during the current study is available from the corresponding author on reasonable request.

### Competing interests

The authors have no conflicts of interest to declare.

### Author contributions

(I) Conception and design: All authors.

(II) Collection and assembly of data: Wen-zhi Xu.

(III) Data analysis and interpretation: Li-qun Meng, Ling-yan Zhang.

(IV) Manuscript writing: All authors.

(V) Final approval of manuscript: All authors.

### Ethics approval

Not applicable.

### Consent to participate

Not applicable.

### Funding

No funding was received for conducting this study.
